# Interactions between the developmental and adult social environments mediate group dynamics and offspring traits in *Drosophila melanogaster*

**DOI:** 10.1038/s41598-017-03505-2

**Published:** 2017-06-15

**Authors:** Juliano Morimoto, Fleur Ponton, Ilona Tychsen, Jason Cassar, Stuart Wigby

**Affiliations:** 1 Department of Zoology, Edward Grey Institute, University of Oxford, South Parks Road, Oxford, OX1 3PS United Kingdom; 2 Charles Perkins Centre, The University of Sydney, Sydney, NSW 2006 Australia; 3 Programa de Pós-graduação em Ecologia e Conservação, Federal University of Paraná, 19031, Curitiba, CEP: 81531-990 Brazil; 4 Department of Biological Sciences, Macquarie University, North Ryde, NSW 2109 Australia

## Abstract

Developmental conditions can strongly influence adult phenotypes and social interactions, which in turn affect key evolutionary processes such as sexual selection and sexual conflict. While the implications of social interactions in phenotypically mixed populations at the individual level are increasingly well known, how these effects influence the fate of groups remains poorly understood, which limits our understanding of the broader ecological implications. To address this problem we manipulated adult phenotypes and social composition in *Drosophila melanogaster* – by experimentally manipulating the larval density of the group-members – and measured a range of group-level outcomes across the lifespan of groups. Adult groups composed of exclusively low larval-density individuals showed high courtship levels, and low early reproductive rates, group growth rates, offspring mass and offspring eclosion success, relative to high larval-density or mixed larval-density groups. Furthermore, high larval-density groups had lower survival. Offspring mass increased with time, but at a reduced rate in groups when male group members (but not females) were from a mixture of larval-densities; peak reproductive rates were also earlier in these groups. Our results suggest that that variation in developmental conditions experienced by adult group members can modify the reproductive output of groups.

## Introduction

In natural populations, varying availability of resources in the environment generate variation in phenotypes between individuals, who then interact in social groups^1, 2^. The pool of resources acquired by organisms is often referred to as the organism’s condition^3^, and all traits are expected to depend on the allocation of resources^4, 5^. Resource allocation modulates the expression of male and female sexually-selected traits that in turn influence key evolutionary processes such as sexual conflict and sexual selection^3, 6–11^. These processes can potentially influence speciation and extinction rates and thus shape biodiversity^12–17^.

Resource acquisition and competition experienced during the developmental stage are known to modulate the social interactions of individuals with their potential mates and rivals in adulthood, both in laboratory and field populations^6, 11, 18–20^. In insects, the developmental environment influences adult body size^21–24^, which tends to positively correlate with female fecundity (i.e. large females produce more eggs than small females) and male competiveness^25^. Large individuals tend to have large reproductive organs, high courtship activity (in males), high mating frequency, and high reproductive output compared to small individuals^6, 7, 21–23, 26–31^. Thus, adult body size is expected to be under fecundity selection in females and sexual selection in males^7, 25, 30, 32–34^.

Most studies on the developmental environment have focused on the plastic responses of focal individuals (e.g. refs 6 and 35), and only recently have studies in *Drosophila melanogaster* begun to investigate the dynamics within mixed-phenotype social groups^7, 11, 36^. In *D. melanogaster*, high larval density, a key ecological factor (e.g. refs 18, 23 and 24), generally results in smaller body-size adults: males that are less competitive with rivals, and females that produce fewer offspring^6, 7, 21, 22^. However, the magnitude of these larval density effects on adult reproduction can be mitigated in mixed-phenotype social groups^7, 36^. Males direct their courtship efforts preferentially to large females in mixed female size environments, which in turn reduces the fecundity advantages of large over small females^36^. Moreover, variation in female body size can affect male siring success whereby large males sire a higher proportion of offspring than small males when females are large, but this effect is lost when females vary in body size^7^.

Despite these observations, it is not straightforward to infer the broad ecological impacts of the interactions amongst phenotypically distinct individuals at the group-level, because the consequences of social interactions within groups are not fully understood (e.g. ref. 37). Key questions remain unanswered: for example, we do not know whether interactions between ecological factors (e.g. larval density, nutrition) and social environments influence the survival and reproductive output of groups and populations. Neither do we understand whether any potential effects are transferred onto subsequent generations, which could further influence group productivity and persistence. This is unfortunate because ecological and social conditions are known to influence important physiological and behavioural processes, including modulation of pathogen transmission and immune responses (e.g. refs 38–41), mating behaviour and reproductive output (e.g. refs 42 and 43), and offspring quality (e.g. refs 44–46). Consequently, the interaction between developmental environments and adult social environments could shape the dynamics between individuals within groups or populations, and influence the likelihood of populations persisting, expanding, or going extinct. Hence, understanding how ecological and social factors interact to modulate group and population fitness not only advances our knowledge of key evolutionary processes, but also has implications for applied biology. For example, factors influencing group productivity and survival are key to species management programs such as for the conservation of endangered populations, and important for the control of agricultural pest species and disease vectors^47, 48^.

Here, we investigate how environmental and social factors influence group-level traits. We first manipulated the ecological environment during development of the fruit fly *D. melanogaster* by changing the larval density, which resulted in differences in the body size in both sexes. We then experimentally combined adults from different larval density developmental environments (i.e. flies of different adult body size), to create adult social environments that varied in the composition of male or female, or both sexes, phenotypes. We measured courtship behaviour levels, reproductive output and survival of these groups, as well as effects on offspring eclosion success and offspring body size. Our study allowed us to gain insight into how ecological and social environments at the larval and adult stages, respectively, interact and how this interaction translates into group-level responses. To our knowledge, this is the first study to explore effects of developmental and social environments at the group-level.

## Predictions

Our predictions were:

1. If males adopt fixed strategies based on their own developmental environment, groups with large males (from low density larval environments) will tend to have higher courtship levels because large males are more sexually active than small males;
2. If females adopt fixed reproductive strategies based on their own developmental environment, groups with large females (from low density larval environments) will tend to have higher offspring production because female body size is positively correlated with female productivity. Moreover, large females should have more resources to expend on provisioning eggs, which might benefit offspring.
3. However, if male and female reproductive traits respond to changes in the phenotype of their social rivals and mates over and above the strategies set by their own developmental environment, then we will expect to see deviations from the patterns predicted above.

## Material and Methods

### Fly stocks and culture

We used wild-type inbred *OregonR* stock of *D. melanogaster* from Bloomington Drosophila Stock Centre and kindly provided to us by Greg Neely’s Lab at the University of Sydney. The stock was maintained in populations (>5,000 individuals) in cages with overlapping generations for >10 generations. All fly stocks were maintained and all experiments conducted, at 25 °C on a 14:10 light:dark cycle in a controlled humidified room (humidity = 68%) and fed with standard sugar-yeast-maize-molasses medium with excess live yeast granules (see Supplementary Information for recipe).

### Larval density manipulation and adult body size

**Figure 1:**
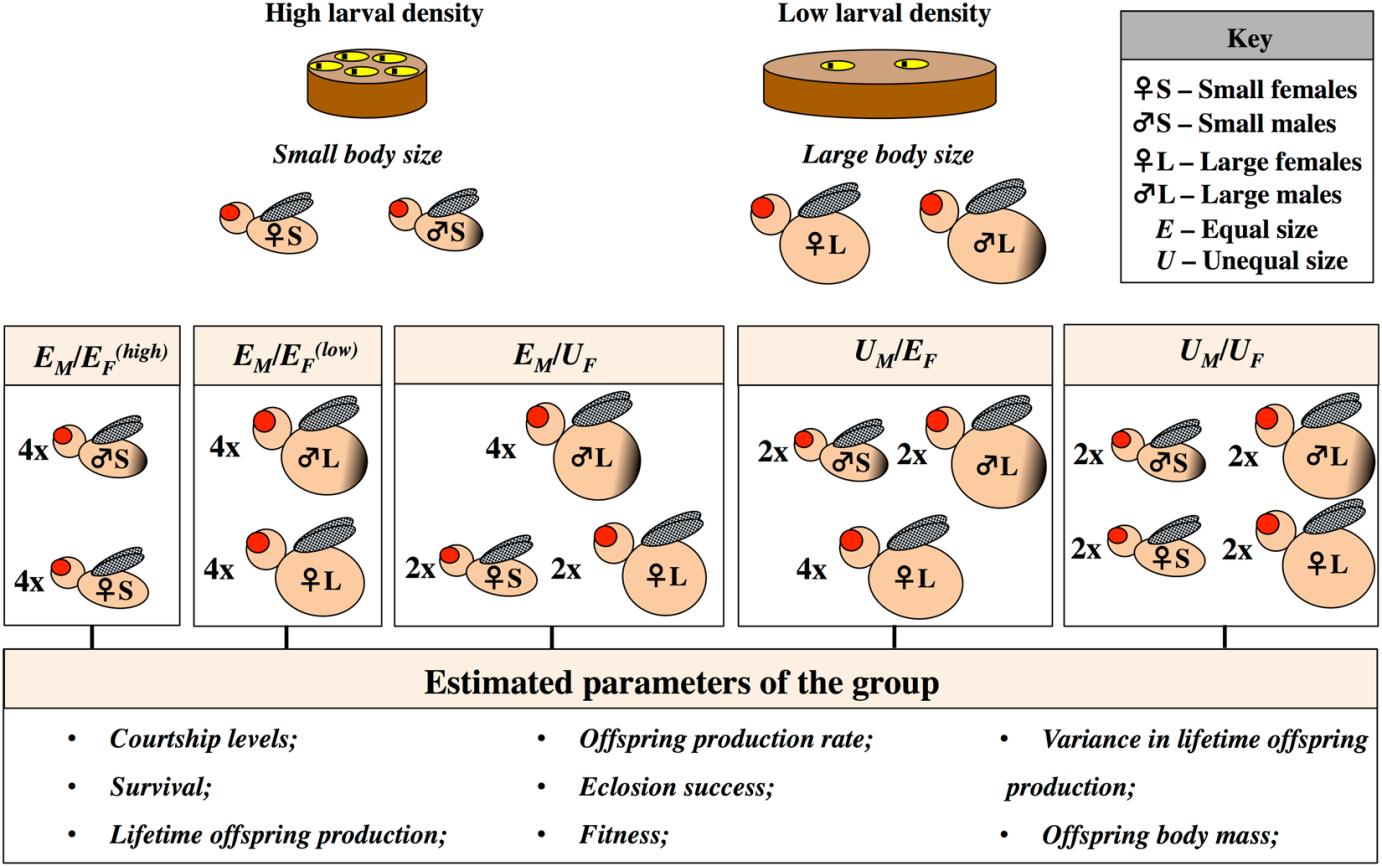
Summary of the experimental design. We manipulated larval density to obtain males and females of large and small body size. We then set up groups composed of individuals with different combinations of body size. E_M_/${{\rm{E}}}_{{\rm{F}}}^{({\rm{high}})}$ – groups where all individuals experienced high larval density; E_M_/${{\rm{E}}}_{{\rm{F}}}^{({\rm{low}})}$– groups where all individuals experienced low larval density; E_M_/U_F_ – groups where all males were phenotypically equal, reared at low density and females had mixed phenotypes; U_M_/E_F_ – groups where all females were phenotypically equal, reared at low density, and males had mixed phenotypes; U_M_/U_F_ – groups where both males and females had mixed phenotypes.

Following the protocol of Clancy and Kennington^49^, we collected eggs from population cages and manipulated larval density of flies in the following way: for the high larval density treatment (small body size adults) we placed ~50 larvae/mL of food (~200 larvae per 34 ml vial containing ~4 mL fly food); for the low larval density treatment (large body size) we placed ~4 larvae/mL of food (~40 larvae in per 34 ml vial containing ~10 mL fly food). A similar approach has been extensively used to create significant differences in the average body size of males and females^6, 7, 21^. We used these categorical variations in body size to manipulate our group social composition (see Fig. 1). Virgin flies of both sexes were collected within 8 hours of eclosion and kept in vials of same-sex and same-larval manipulation groups of 15–20 individuals for 2–5 days prior to experiments. We used >50 vials per treatment to rear flies. Flies form these vials were pooled within each larval density treatment, before being randomly allocated to each adult group treatment. This methodology reduced the likelihood of placing individuals that had been reared in the same vials in the same treatments.

### Experimental design

We created groups with ‘Equal’ (E) or ‘Unequal’ (U) phenotypes of male (M), female (F), or both (Fig. 1). The design was based on Morimoto *et al*.^7^. Briefly, replicate groups containing 4 flies of each sex were placed in vials (i.e. 1 group of 8 flies per vial), and flies were allowed to interact continuously throughout their lifespan. Groups were transferred to fresh vials with standard maize-molasses food and yeast *ad libitum* in discrete time intervals (i.e. days 3, 6, 9, 13, 16, 19, 23, 27, 35, 40, 45 and 50) throughout their lifespan. We assembled 17 replicate groups *per* treatment, in five treatments (see Fig. 1):

1. Groups with equal-sized males and equal-sized females in which males and females experienced high larval density (small adult body size) (E_M_/${{\rm{E}}}_{{\rm{F}}}^{({\rm{high}})}$);
2. Groups with equal-sized males and equal-sized females, in which males and females experienced low larval density (large adult body size) (E_M_/${{\rm{E}}}_{{\rm{F}}}^{({\rm{low}})}$);
3. Groups with phenotypically equal-sized males but unequal-sized females, in which all 4 males experienced low larval density, 2 females experienced high and 2 females experienced low larval density (E_M_/U_F_);
4. Groups with phenotypically unequal-sized males but equal-sized females, in which 2 males experienced high larval density and 2 males experienced low larval density, all 4 females experienced low larval density (U_M_/E_F_);
5. Groups with phenotypically unequal-sized males and unequal-sized females (U_M_/U_F_).

### Courtship

Courtship levels in groups were assessed based on the sum of the number of male courtship behaviours towards females (e.g. chasing, wing-extension, attempting copulation^50^) that were observed every 15 min for 1 h. These observations were taken on the mornings of days before groups were transferred to fresh vials (see above). This allowed us to estimate the courtship levels of the group over time. After the behavioural observations of courtship, we transferred the groups to vials with fresh standard maize-molasses food and yeast *ad libitum* until the next vial replacement.

### Survival

We scored deaths and calculated the number of males and females alive in each group at each time interval used for the behavioural and reproductive measurements. Group survival was defined as the survival of the 4 adult females in the group, in which case males, if still alive, were discarded.

### Offspring number

After fly removal, we stored all interaction vials for 13–15 days, until all the adult offspring had emerged. We scored the number of eclosing offspring produced in each vial and calculated the rate of offspring production and total offspring production of each group. We also scored the number of non-emerging pupae to measure eclosion failure. We scored courtship and offspring for the first 35 days, by which time females had stopped producing any viable eggs (i.e. no more offspring). From this point on we only scored survival.

### Offspring mass

We randomly sampled ~25 female and ~25 male offspring *per* group *per* day (*N*_*total*_ = 1*,458*) and measured their wet body mass on a fine scale Sartorius® with precision of 0.001 g.

### Data analysis

#### Larval density effects on body size

We used one-way ANOVA after checking for the normality of the data to evaluate the effects of larval density on male and female body sizes.

### Courtship

To analyse courtship we used a Generalized Linear Model (GLM) with a Poisson distribution and accounted for overdispersion of the data using a *quasi* extension. The model included the linear and quadratic effects of time, treatment and their interactions. As individuals died, they were not replaced and thus the number of individuals contributing to group fitness and survival declined. To control for this, we included covariates in the models to account for the number of females and males in the group at the time of the courtship activity measurements. We also included vial to control for pseudoreplication. p-values are given from F-tests. We used Student-Newman-Keuls (‘SNK’) post-hoc test to determine differences in mean courtship levels between groups.

### Survival

We tested for differences in survival of the group by fitting a Cox proportional hazards model using the ‘coxph’ function of the ‘survival’ package in R^51^; p-values are given from the χ^2^ test.

### Offspring production

To analyse offspring production, we fitted a general linear model with a normal error distribution on the full dataset that included – in addition to the variables described above – the interactions between the linear and quadratic effect of time and treatment (i.e. Time*Group and Time^2^*Group). This allowed us to compare how the slope and the peak reproductive success of groups differ depending on the phenotype composition of males and females in the group. For the group total offspring production, we used SNK post-hoc test to check for differences between treatments. We checked for the normality and the homogeneity of variances (df = 4, Bartlett’s K-squared = 2.695, p = 0.610) of the data for the general linear model.

### Group fitness

We estimated group growth rates by calculating *r*, an index obtained from the age-specific offspring production and survival, and which its estimates better represent fitness than lifetime offspring production in *D. melanogaster* (see refs 33, 52 and 53) (see Supplementary Information for details). Higher *r* estimates represent higher fitness and vice-versa^33, 52, 53^. We calculated *r* from each group (see Supplementary Information), and tested differences between treatments with a non-parametric Kruskal-Wallis Rank Sum Test (with p-values reported form a ${\chi }^{2}$ test) followed by a Student-Newman-Keuls (SNK) post-hoc test to assign differences in means.

The timing of reproduction has a large impact on population growth rates. We therefore investigated whether social and developmental environments interact to alter age-specific group reproductive rates. To do this, we fitted general linear models for each treatment individually, which included linear and quadratic effects of time while controlling for the number of females and males in the group as described above. When the quadratic effect of time was statistically significant, we calculated the peak reproductive success of the group with the derivative of the quadratic model as follows:

1 $$F(x)=\alpha {x}^{2}+\beta x+\gamma $$

where *α* and β are the quadratic and linear coefficients of the linear model, respectively, after controlling for confounding covariates and *x* is our time intervals. We then took a derivative of eq. (1)

2 $$\frac{\partial F(x)}{\partial x}=2\alpha x+\beta $$

where $\frac{\partial F(x)}{\partial x}$ is the first derivative of eq. (1) in time. If *α < 0*, then solving eq. (2) for $\frac{\partial F(x)}{\partial x}=0$ defines the point in which eq. (1) is maximised – i.e. it defines the day when the rate of offspring production peaked.

We also investigated whether the variance in group total reproductive success was affected by our treatments using the ‘leveneTest’ function of the ‘car’ package in R^54, 55^, which allowed us to test for homogeneity of variance between our treatments. We calculated Pearson’s correlation to investigate whether overall harassment levels correlated with overall reproductive success.

### Pupal eclosion

To analyse the proportion of non-emergent pupae, we used a generalized linear model with a Binomial distribution corrected for overdispersion with the *quasi* extension, with treatment as the main factor while controlling for the total number of pupae in the group (i.e. adult offspring (emergent pupae) + non-emergent pupae). Courtship level was also included as a covariate in the model to investigate whether courtship levels affected eclosion failure; p-values are given from F-tests. We used the SNK post-hoc test to test for differences in the mean non-emergent pupae between our treatments.

### Offspring body mass

To test for effects on offspring body mass we used a general linear model with a normal distribution of errors. The independent variables in the model were treatment, sex of the offspring and their interaction, vial, courtship, the density of offspring per vial and the linear and quadratic effects of time and their interaction with treatment. We Boxcox transformed offspring mass (i.e. mass^0.5) to fit the normality assumption. We used the SNK post-hoc test to test for differences in mean offspring body mass between our treatments.

All independent variables in our models were established a priori and thus no model simplification was applied. Figures are of non-transformed (raw) data plotted with the package ‘ggplot2’^56^. Thick lines in figures were created with function “loess”, available within the ‘ggplot2’ package^56^ (see Supplementary Information for details). All analyses were performed in R version 3. 2. 2^54^.

## Results

### Larval development manipulation and body size

Males and females from high and low larval density treatments had significantly different body sizes (*Male:* F_1,68_ = 18.336, p < 0.001; *Female:* F_1,74_ = 14.725, p < 0.001) whereby individuals from high larval density had smaller body size than individuals from low larval density treatments (Fig. S1) [*Sex: Treatment: Mean* ± *SE*; Male: *Low larval density:* 0.658 ± 0.010, *High larval density:* 0.588 ± 0.012; Female: *Low larval density:* 0.792 ± 0.013, *High larval density:* 0.719 ± 0.013]. The variance in body size was not affected by our larval manipulation in either males (F_var36,33_ = 0.806, p = 0.526) or females (F_var36,39_ = 1.006, p = 0.979).

### Group survival

**Figure 2:**
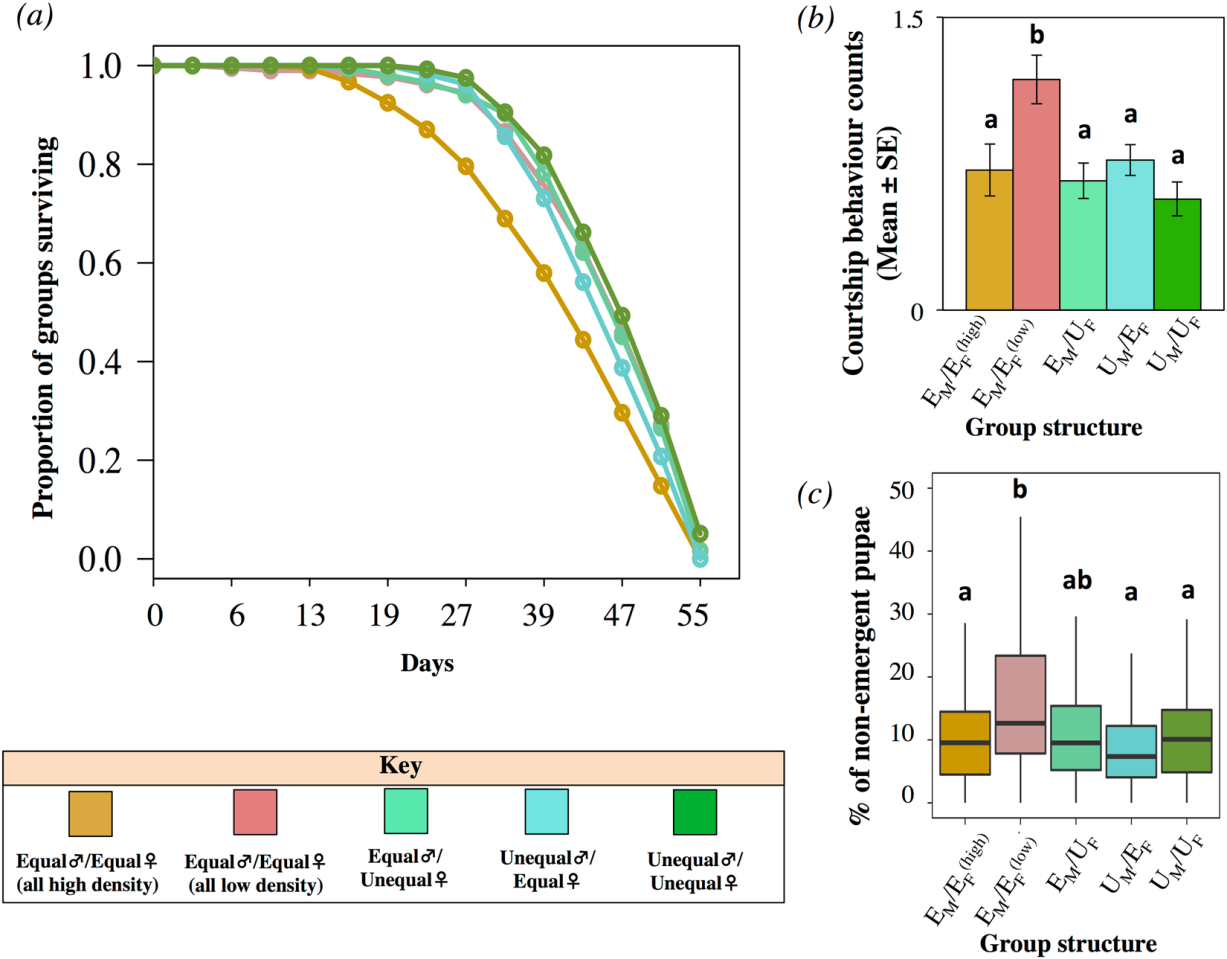
Survival, courtship levels, and pupae eclosion. (**a**) Proportion of groups surviving throughout the experiment. Likelihood ratio test = 20.74, df = 5, p < 0.001. (**b**) Courtship levels in the treatments. (**c**) Pupae eclosion success (in %) measured as the number of non-emergent pupae. Post-hoc SNK test (α = 0.05).

Treatment had a significant effect on survival (Treatment: χ^2^_4_ = 20.660, p < 0.001). Groups in which both males and females experienced high larval density (i.e. E_M_/${{\rm{E}}}_{{\rm{F}}}^{({\rm{high}})}$) had significantly lower survival than groups in which both males and females experienced low larval density (i.e. E_M_/${{\rm{E}}}_{{\rm{F}}}^{({\rm{low}})}$), suggesting that high larval density can shorten group lifespan. There was no difference in survival between U_M_/E_F_, E_M_/U_F_ and U_M_/U_F_ treatments relative to E_M_/${{\rm{E}}}_{{\rm{F}}}^{({\rm{low}})}$ (Fig. 2a).

### Courtship levels

Groups in which both males and females experienced low larval density (i.e. E_M_/${{\rm{E}}}_{{\rm{F}}}^{({\rm{low}})}$) had significantly higher courtship behaviour counts than all other groups which did not significantly differ from one another (F_4,527_ = 4.962, p < 0.001, Fig. 2b). There was no evidence for a linear nor a quadratic effect of time (Time: F_1,531_ = 1.468, p = 0.226, Time^2^: F_1,526_ = 0.871, p = 0.351, Table S1).

### Pupae eclosion

Treatment had a significant effect on the proportion of non-emergent pupae (F_4,489_ = 8.395, p < 0.001; see Table S2). Groups in which both males and females experienced low larval density (i.e. E_M_/${{\rm{E}}}_{{\rm{F}}}^{({\rm{low}})}$) had significantly more non-emergent pupae than treatments E_M_/${{\rm{E}}}_{{\rm{F}}}^{({\rm{high}})}$, U_M_/E_F_, U_M_/U_F_, and a non-significant trend in the same direction for E_M_/U_F_ (Fig. 2c). This effect was above and beyond the effects of offspring density in each vial, since the contribution of offspring density within vials was accounted for in the statistical model (F_1, 492_ = 31.624, p < 0.001). In addition, these effects were independent of courtship levels because there was no significant correlation between courtship levels and pupae eclosion (F_1, 486_ = 0.715, p = 0.398, Table S2).

### Total offspring production

**Figure 3:**
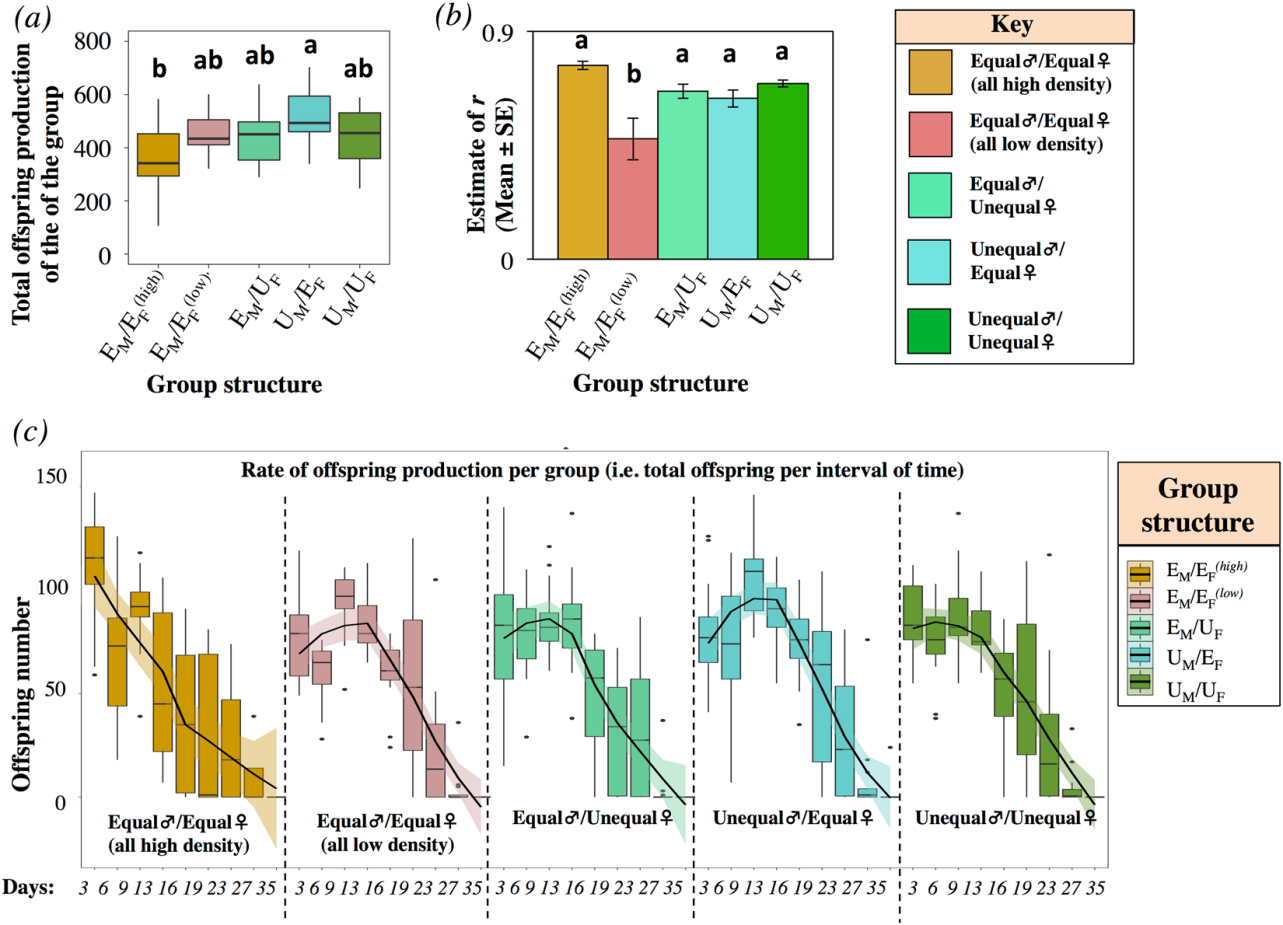
Estimates of group reproductive success, fitness, and offspring production rate. (**a**) Total lifetime offspring production. (**b**) Age-specific fitness of groups (*r*). (**c**) Offspring production *per* interval of time. Post-hoc SNK test (α = 0.05).

Treatment had a significant effect on total offspring production (F_4,75_ = 4.017, p = 0.005, Table S3). A post-hoc analysis revealed that groups in which males and females experienced high larval density (i.e. E_M_/${{\rm{E}}}_{{\rm{F}}}^{({\rm{high}})}$) produced significantly fewer offspring than U_M_/E_F_ groups, and all other groups were intermediate and not significantly different from one another (Fig. 3a).

### Group fitness, r

We calculated *r*, an age-specific offspring production index often used as a proxy for group fitness (see e.g. refs 33, 53 and 57). Treatment had a significant effect on estimates of *r* (Treatment: ${\chi }_{4}^{2}$ = 23.191, p < 0.001) whereby groups composed of males and females from low larval density developmental environment (i.e. E_M_/${{\rm{E}}}_{{\rm{F}}}^{({\rm{low}})}$) had significantly lower fitness estimates (i.e. lower estimates of *r* [see Methods]) than other treatments (Fig. 3b).

### Offspring production rate

Time had a significant negative linear and quadratic effect on the rate of offspring production of the groups (Time: F_1, 607_ = 714.652, p < 0.001; Time^2^: F_1, 607_ = 11.354, p < 0.001, Fig. 3c, Table S3), which was expected since females reduce (and eventually stop) producing eggs through time. There was no significant interaction between linear Time and Group (F_4, 607_ = 1.238, p = 0.293). However, we found a significant quadratic interaction, Time^2^*Group, on offspring production (F_4,607_ = 4.540, p = 0.001, Fig. 3c), suggesting that the peak reproductive success differed between groups. Relative comparison between treatments revealed that this effect was mainly driven by groups where males and females were small (i.e. E_M_/${{\rm{E}}}_{{\rm{F}}}^{({\rm{high}})}$), in which the estimates of the concavity of the curve did not reach statistical significance (Time^2^: *t-value* = *0.462*, p = 0.645). This pattern suggests that E_M_/${{\rm{E}}}_{{\rm{F}}}^{({\rm{high}})}$ reached maximum reproductive success between days 0–3, after which reproduction declined linearly (Fig. 3c). The number of males and females in the group at the time of the measurement was significantly positively correlated with the rate of offspring production (see also Table S3 and Fig. S3 for cumulative offspring production).

We then used eqs (1) and (2) to calculate the peak of the rate of offspring production in our groups. For groups with males from low larval density environments (E_M_/${{\rm{E}}}_{{\rm{F}}}^{({\rm{low}})}$ and E_M_/U_F_) the estimates of peak reproductive success laid in between days 6–9 of group survival, whereas groups with mixed male phenotypes (i.e. U_M_/E_F_ and U_M_/U_F_) reached peak reproductive success at around days 9–13. For groups with males and females from high larval density developmental environments (E_M_/${{\rm{E}}}_{{\rm{F}}}^{({\rm{high}})}$) the peak reproductive success was the earliest among our treatments (0–3 days) (Fig. 3c, see Table S4).

### Offspring body mass

**Figure 4:**
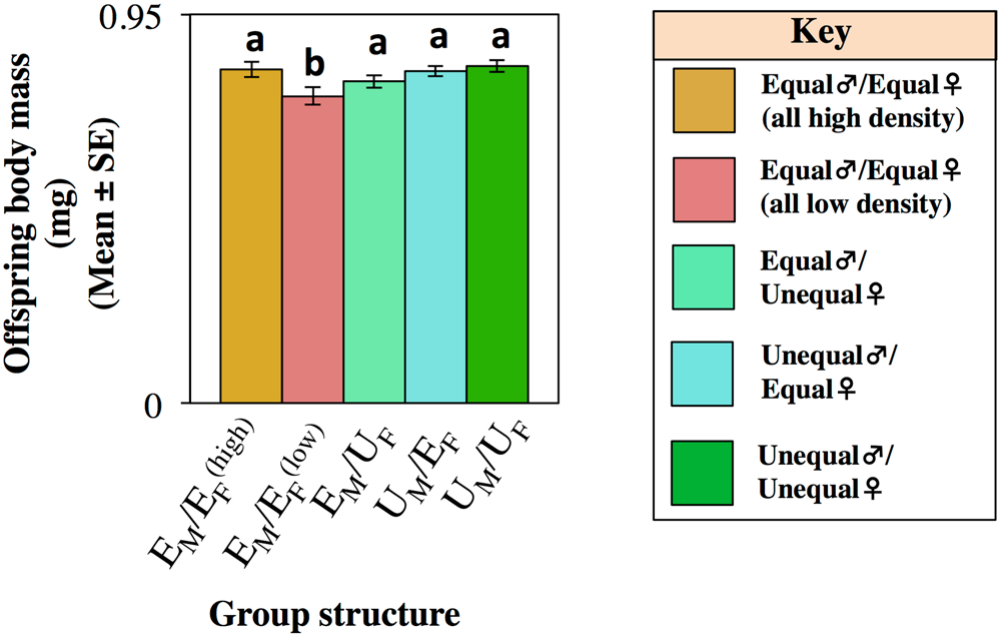
Offspring body mass. Average offspring body mass (in mg) (Mean ± SE). Post-hoc SNK test (α = 0.05).

Group had a significant effect on offspring weight (F_4,329_ = 7.042, p < 0.001). Post-hoc analysis revealed that this effect is caused by a reduction in offspring weight in groups composed of all large male and female individuals (i.e. E_M_/${{\rm{E}}}_{{\rm{F}}}^{({\rm{low}})}$) compared to all other treatments (Fig. 4, Table S5). There was a linear and a quadratic effect of Time (Time: F_1,329_ = 162.326, p < 0.001; Time^2^: F_1,329_ = 157.184, p < 0.001) suggesting that offspring body mass increased after the first day of egg-laying and plateaued after ~9–13 days after the beginning of the experiment. There was a significant interaction of Group*Time and Time^2^ (Group*Time: F_1,329_ = 6.979, p < 0.001; Group*Time ^2^ *:* F_1,329_ = 3.332, p = 0.010), which showed that the increase in offspring body mass differed between groups (Fig. S4; Table S5). Comparison of the slopes of offspring body mass on time revealed that the slope of U_M_/U_F_ and the U_M_/E_F_ groups increased at a slower rate than E_M_/${{\rm{E}}}_{{\rm{F}}}^{({\rm{low}})}$, although this effect was not found for E_M_/${{\rm{E}}}_{{\rm{F}}}^{({\rm{high}})}$ and E_M_/U_F_ groups. These data indicate that in groups with mixed male phenotypes the body mass of offspring increases a slower rate over time. Courtship levels were not correlated with offspring body mass (F_1,329_ = 1.389, p = 0.239), suggesting that the reduction in offspring body mass was not driven by group courtship levels. The interaction Sex*Treatment was not significant (Sex*Treatment: F_4,329_ = 1.395, p = 0.235).

## Discussion

The data presented here show that phenotypically homogeneous groups where individuals of both sexes experienced high larval density (i.e. E_M_/${{\rm{E}}}_{{\rm{F}}}^{({\rm{high}})}$) had high early reproductive rates (see *r*) and offspring eclosion success, but group survival was lower relative to other treatments. When both males and females experienced low larval density (i.e. E_M_/${{\rm{E}}}_{{\rm{F}}}^{({\rm{low}})}$), groups had low early reproductive rates (see *r*), produced offspring with lower body mass and lower eclosion success, which were rescued in groups consisting of mixed phenotypes. In groups where males, but not females, were phenotypically mixed, there was a delayed peak in reproduction and reduced rates of increasing offspring body mass. Overall, these results suggest far-reaching effects of the interaction between developmental and social environments on offspring and group-level traits. Below, we discuss the main findings of our study in more detail.

### Courtship levels and trans-generational effects

We found that groups composed of males and females from the low larval density developmental environment (large body size phenotype) had higher courtship levels (Fig. 2b) and produced offspring with lower body mass (Fig. 4). Moreover, we showed that the presence of some individuals from high larval density (i.e. phenotypically mixed groups) was sufficient to lower courtship levels and increase offspring body mass. These results contrast with predictions 1 and 2 in which we would expect courtship to be higher in all groups consisting of males from the low larval density developmental environment (i.e. E_M_/${{\rm{E}}}_{{\rm{F}}}^{({\rm{low}})}$ and E_M_/U_F_) (prediction 1), and offspring body mass to be significantly higher in all groups consisting of females also from the low larval density environment (i.e. E_M_/${{\rm{E}}}_{{\rm{F}}}^{({\rm{low}})}$ and U_M_/E_F_) (prediction 2). Thus, the data suggest prediction 3 to be true: the social and developmental environments interact to shape courtship activity and offspring traits. There are several possible explanations for our findings. Female phenotypic variation may trigger male adaptive choice, whereby males direct their courtship efforts towards large and more attractive females^36, 58^. Previously, Long *et al*.^36^ have shown that the decrease in courtship effort towards small females is greater than the increase in courtship effort towards large females. Thus, the female phenotypic variation in our experiment could have trigger male adaptive response and led to a reduction on the average courtship level of the group. Moreover, female phenotypic variation may also increase the uncertainty of females in assessing the level of intra-sexual competition for resources, which may trigger female responses to increase investment in their progeny in order to guarantee their offspring’s success in the next generation of unpredictable competition. In parallel, male phenotypic variation is also likely to have influenced group dynamics. In groups consisted of mixed male phenotypes, large males (from low larval density) might have perceived weaker intra-sexual competition (due to the presence of small males), and therefore may decrease their courtship investment to allocate resources in ejaculate and offspring health. Furthermore, the larval manipulation could have signalled the level of intra-sexual competition that individuals were likely to face in adulthood^19, 20^ which in turn could have altered male and female offspring resource allocation ^59, 60^ and life-history strategies^33^. Scrutinising these hypotheses lies beyond the scope of this paper, but remains an important topic of research for future studies.

We also found that offspring body mass increased with group age and plateaued at ~9–13 days of group survival, which was approximately coincident with peak reproductive success (except for E_M_/${{\rm{E}}}_{{\rm{F}}}^{({\rm{high}})}$, see discussion below) (Fig. S4). Although the significance of this pattern is unclear, differential female offspring allocation and male accessory gland maturation could have influenced offspring body mass^60–62^. In *Daphnia magna*, maternal age is positively associated with offspring size^63^. On the other hand, female age delays development and is negatively associated with egg size and hatchability in *Collosobruschus maculatus* ^64^. In *D*. melanogaster, the quantity of seminal fluid and the size of male accessory gland increase with male age^62^, which could alter male ejaculate investment and account, at least partly, for the increased offspring body mass with increased population age. Moreover, theory suggests that maternal investment in offspring might increase with age^65^ although evidences of this effect in *D. melanogaster* are still lacking. More studies are needed to investigate the significance of the increase in offspring body mass as the population aged.

### Group reproductive output and fitness

If the developmental environment was the sole factor determining group fitness, we would have observed higher group fitness for groups consisting of females reared at low density (i.e. E_M_/${{\rm{E}}}_{{\rm{F}}}^{({\rm{low}})}$ and U_M_/E_F_) (prediction 2) because these females are larger and more fecund. However, our results suggest that groups consisting of males and females from low larval density developmental environments (i.e. E_M_/${{\rm{E}}}_{{\rm{F}}}^{({\rm{low}})}$) had lower estimates of group fitness *r*, which was fully rescued in phenotypically mixed groups of either (or both) sex(es). (Fig. 3a and b). These findings corroborate prediction 3, and shows that the interaction between the developmental and social environments shapes group fitness (see ‘Predictions’). We also showed that the peak reproductive success for groups composed of males from low larval density was earlier (6–9 day of survival) than for groups with mixed male phenotypes (9–13 day of survival). Social and developmental environments can potentially modulate group reproductive success and fitness by altering female reproductive investment as a response to social context as well as male ejaculate investment based on competition levels or accessory gland maturation, or both^62, 66^. We also found that groups consisting of males and females from high larval density (i.e. E_M_/${{\rm{E}}}_{{\rm{F}}}^{({\rm{high}})}$) had the earliest peak reproductive success amongst our treatments (0–3 days of group survival), and group reproduction rate became a negative linear function in time (Fig 3c). This pattern might arise from possible differences in life-history strategies at the individual level caused by social interactions, whereby males from high larval density environments, when surrounded by rivals with the same background experiences of stressful developmental environment, adopted a strategy to invest heavily on current reproduction at the expense of survival and future reproduction^5, 33, 67^. Future studies should investigate the relationship between larval density and male maturation as well as individuals’ strategies (as opposed to groups’ responses) adopted in different social contexts.

### Group survival

Groups that consisted of males and females from high larval density environments (small body size) died faster than other treatments (Fig. 2a), corroborating previous findings that small individuals have shorter lifespan^26, 68^. Phenotypically mixed groups of either males or females, or both, showed similar survival to groups consisted of individuals from low larval density environments, which supports our prediction 3 that social and developmental environments also interact to shape group survival (see discussion above). Had the developmental environment been the sole factor affecting group survival, we might expect groups consisted of males from low larval density environment to die more rapidly because of the higher costs incurred by the elevated courtship activity of these males (prediction 1). Instead, group survival was likely to have been influenced by a myriad of traits of an individual (e.g. female reproductive output) and the interaction between traits among individuals (e.g. female post-mating responses to male seminal fluid proteins) and therefore, further studies are required to fully comprehend the survival patterns observed here. Interestingly, however, Adler *et al*.^69^ recently showed that the developmental and social environments modulate somatic deterioration of males neriid fly *Telostylinus angusticollis*, whereby somatic deterioration is significantly elevated for high condition males in social groups to the point in which high condition males did not outlive low condition males. Together, our results and the results of Adler *et al*.^69^ open a new avenue for future research on the interaction between ecological and social factors affecting group survival.

### Interactions between development and social environments

The plastic responses of different phenotypes to the interactions with their surrounding environment are crucial for the success of individuals in nature^70^. This plasticity may allow individuals to survive and persist in new environments when facing unprecedented challenges, which may in turn influence group-level success^71, 72^. These patterns are revealed in our data whereby plastic responses to social and developmental environments modulates group survival and trans-generational effects in eclosion success and body mass, which might influence long-term group persistence in challenging environments. Plastic responses to developmental and social environments can shape mating and non-mating behaviour of individuals, affecting the distribution, the quality, and the availability of mates and rivals in the group^7, 73–75^. As a result, plastic responses to the developmental and social environments can modulate the operation of sexual selection in groups, which in turn can shape group survival, growth and speciation patterns^7, 72, 76–78^. This study found that courtship levels, reproduction and fitness of groups are determined by plastic responses to social and developmental environments, which lend support to the idea that plastic responses to the surrounding environmental conditions can modulate sexual and reproductive behaviours and ultimately influence key evolutionary processes. For example, by influencing courtship levels, the developmental and social environments likely affect the operation of sexual selection and sexual conflict. High levels of courtship can harm females, and thus represents a potential source of conflict between the sexes. Specifically, *D. melanogaster* populations where females experience high courtship have lower survival and reproductive success, thus potentially reducing their fitness (e.g. refs 12, 36, 58, 79 and 80). Both theoretical and empirical work have suggested that high sexual conflict could influence the potential for populations to undergo speciation or go extinct (e.g. refs 78, 81–83). Therefore, developmental conditions may lead to a cascade of effects via social interactions and behaviour that modulates sexual conflict and affects the success or failure of populations in the long-term. More work is needed to disentangle precisely how ecological and social conditions mediate sexual conflict, and what this means for group-level metrics. Nonetheless, future ecological and evolutionary studies should acknowledge the implications of multi-level interactions between the early-life experiences (e.g. larval density, larval nutrition), and the social context of competing adults, in order to fully comprehend processes such as sexual selection and sexual conflict.

## Conclusion

In conclusion, our results show important effects of the developmental and social environments on group fitness in *D. melanogaster* and add to the growing body of research on environmentally-mediated trans-generational effects. All species face challenges in their developmental environment (i.e. physical or nutritional constraints), and in most species individuals are required to interact during their lifetime both for survival and reproduction. Thus, the patterns revealed here could potentially be widespread, and future studies should expand and test our results and predictions in different species across the animal kingdom. Important questions still remain, such as (1) What are the individual level responses to the interactions between developmental and social environments? (2) What are the mechanisms by which individuals discriminate rivals and mates from similar or different developmental environment? (3) What are the molecular mechanisms by which the developmental and social environments interact and lead to the trans-generational effects observed in this study? (4) What are the long-term fitness consequences for the offspring for having lower body mass? (5) For how many generations do the trans-generational effects of the development and social environment persist? The answer to these questions will advance our understanding of the evolutionary and ecological processes affected by the structure, composition and social dynamics of groups and populations.

### Data accessibility

Data is available at https://doi.org/10.5287/bodleian:vmgkaD2w9.

## Electronic supplementary material

Supplementary Information
