# Supplementary material for: Interactions between the developmental and adult social environments mediate group dynamics and offspring traits in Drosophila melanogaster
Source: Sci Rep. 2017 Jun 15;7:3574. doi: 10.1038/s41598-017-03505-2 (PMC5472581; doi:10.1038/s41598-017-03505-2)
Supplement: Supplementary file 1 — Supplementary Information [file 41598_2017_3505_MOESM1_ESM.pdf]

Supplementary Information: "Interactions between the developmental and adult social environments mediate group dynamics and offspring traits in *Drosophila melanogaster*"

Juliano Morimoto[1,2,3]*, Fleur Ponton[4], Ilona Tychsen[4], Jason Cassar[4], Stuart Wigby[1]

Authors' affiliation:

[1]Department of Zoology, Edward Grey Institute, University of Oxford, South Parks Road, Oxford OX1 3PS, United Kingdom

[2] Charles Perkins Centre, The University of Sydney, NSW 2006, Australia

[3]Programa de Pós-graduação em Ecologia e Conservação, Universidade Federal do Paraná, Curitiba, Brazil

[4] Department of Biological Sciences, Macquarie University, NSW 2109, Australia

*To whom correspondence should be addressed.

E-mail: juliano.morimoto@sydney.edu.au

Supplementary Methods

Calculating *r*

We estimated r, the intrinsic rate of increase of the groups, as

$$w_g = \sum_{x=0}^{wi} e^{-rx}\, l_i(x) \qquad \text{eq. S1}$$

where w_g is the fitness of the group, x is the adult age of first all through last reproduction w of individual i, and l_i is the age-specific product of survival and reproduction (see [1]). Notice that higher (lower) values o r indicates higher (lower) intrinsic rate of increase of the group and hence higher (lower) fitness.

Loess

Loess is a function built in R[2] that refers to Local Regression. Local regression are non-parametric methods that fit a smoothed multiple regression in a local neighbourhood of the data[2]. In out study, the loess function was used to provide visual guidance of the trends in our data.

Supplementary Results

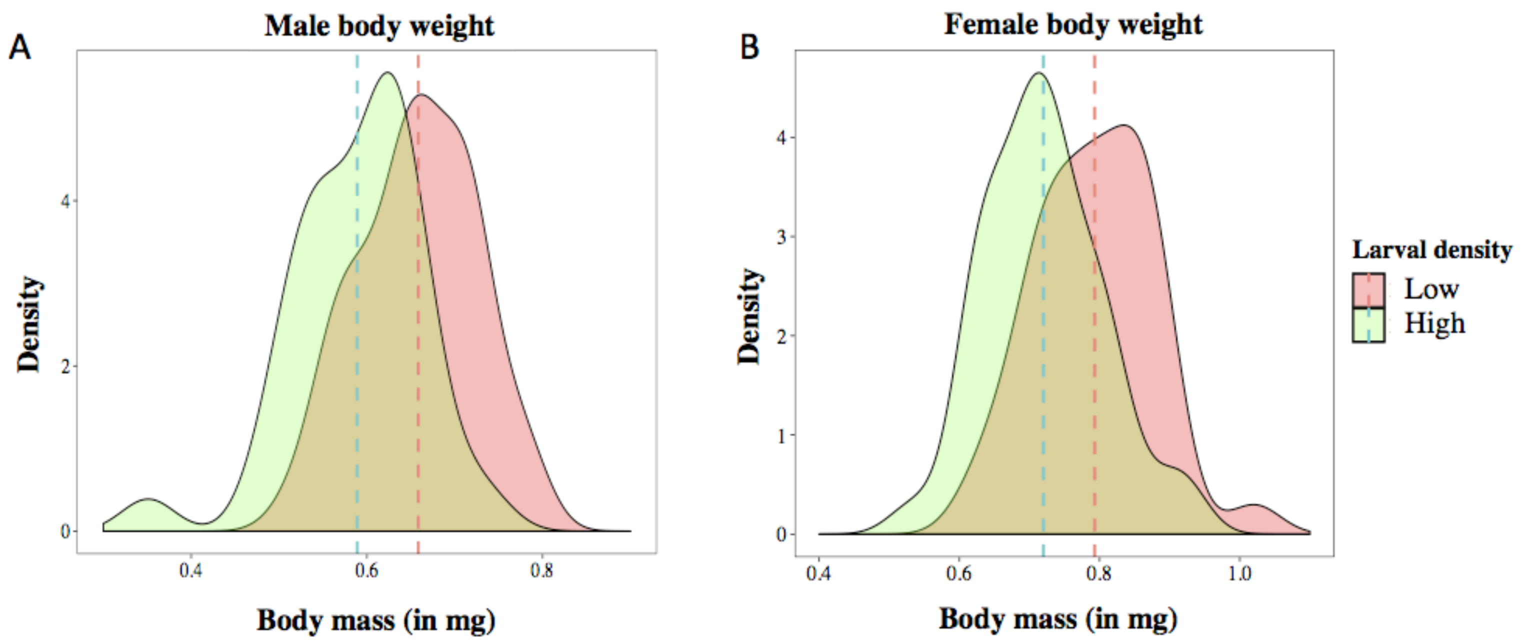

Figure S1 – Differences in body size caused by manipulations in the larval density (in mg). (**A**) Density plot of male weight and (**B**) Density plot of female weight. Green – High larval density; Red – Low larval density. Green dashed line – average body size of males and females raised at high larval density; Red dashed line - average body size of males and females raised at low larval density.

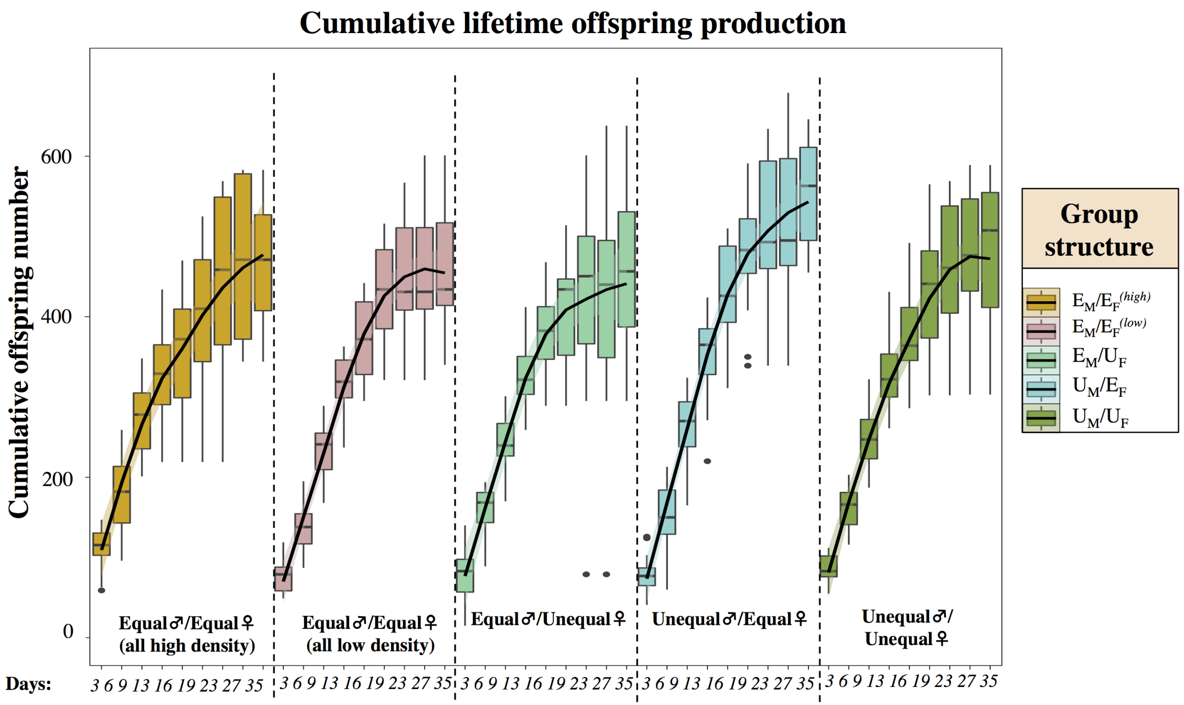

Figure S2 – Cumulative offspring production.

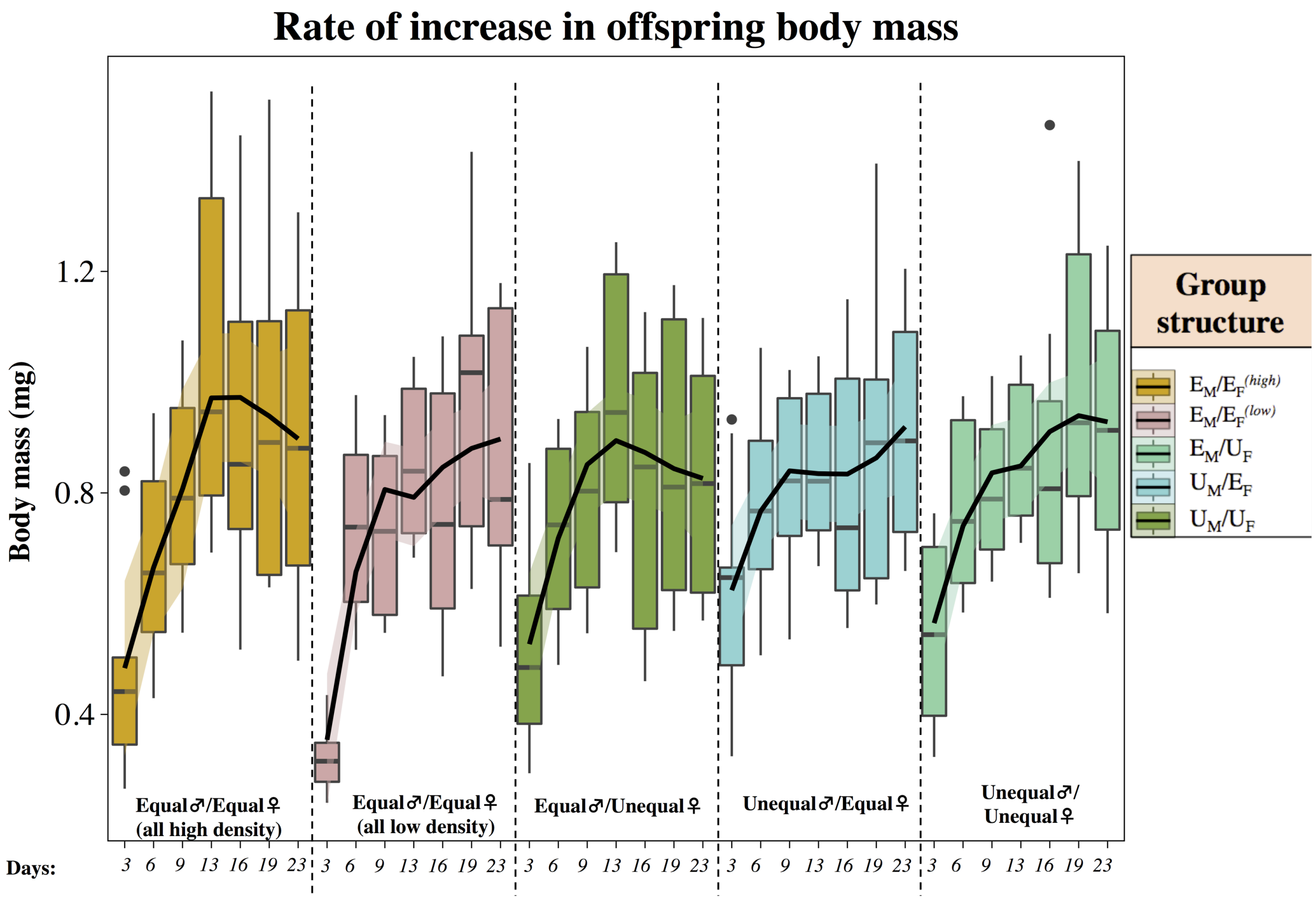

Figure S3 – Rate of increase in offspring body mass (in mg).

Table S1 – Complete analysis of group courtship levels. **Bold:** $p < 0.05$.

**Factors**	**Courtship levels**	
	F-value	***p-value***
**Group**	4.962	**<0.001**
**Time**	1.468	0.226
**Time^2**	0.871	0.351
**Number of Males**	0.312	0.576
**Number of females**	2.845	0.092
**Group*Time**	0.987	0.413
**Group*Time^2**	2.049	0.086
**Vial**	0.445	0.504
Dispersion	*1.406*	

Table S2 – Complete analysis pupae eclosion. **Bold:** $p < 0.05$.

Factor	Pupal eclosion	
	F-value	***p-value***
**Group**	8.395	**<0.001**
**Vial**	0.753	0.385
**Density of individuals**	31.624	**<0.001**
**Courtship levels**	0.715	0.398

Table S3 – Complete analysis of total reproductive success and rate of offspring production. **Bold:** $p < 0.05$.

**Factors**	**Total reproductive success**		**Rate of offspring production**	
	F-value	***p-value***	***F-value***	***p-value***
**Group**	4.017	**0.005**	3.598	**0.018**
**Vial**	0.926	0.338	1.647	0.199
**Time**	-	-	714.652	**<0.001**
**Time^2**	-	-	11.354	**<0.001**
**Number of males**	-	-	22.421	**<0.001**
**Number of females**	-	-	15.211	**<0.001**
**Group*Time**	-	-	1.238	0.293
**Group*Time^2**	-	-	4.540	**0.001**

Table S4– Complete analysis of the effects of time (both linear and quadratic) on offspring production rate within each group treatment, while controlling for the effects of the number of males and females contributing to the group at the time interval. Peak reproductive success was calculated as described in the main text. **Bold:** $p < 0.05$.

Group treatment	Factor								
	Time		$Time^2$		Number of males		Number of females		Peak
	F-value	*p-value*	*F-value*	*p-value*	*F-value*	*p-value*	*F-value*	*p-value*	
$E_M/E_F^{(high)}$,	107.921	**<0.001**	1.997	0.161	5.092	**0.026**	10.089	**0.002**	0-3 days
$E_M/E_F^{(low)}$	155.410	**<0.001**	37.280	**<0.001**	0.733	0.393	0.953	0.330	6-9 days
E_M/U_F	143.590	**<0.001**	14.690	**<0.001**	4.642	0.331	2.686	0.103	6-9 days
U_M/E_F	155.550	**<0.001**	43.602	**<0.001**	2.841	0.094	2.526	0.114	9-13 days
U_M/U_F	245.417	**<0.001**	29,527	**<0.001**	17.013	**<0.001**	3.110	0.080	9-13 days

Table S5 – Complete analysis of the rate of increase in offspring mass. **Bold:** $p < 0.05$.

**Factors**	**Rate of increase in offspring body mass**	
	F-value	***p-value***
**Density of offspring in each vial**	211.313	**<0.001**
**Courtship levels**	1.389	0.239
**Group**	7.042	**<0.001**
**Time**	162.326	**<0.001**
**Time^2**	157.184	**<0.001**
**Vial**	0.114	0.735
**Sex (offspring)**	783.891	**<0.001**
**Group*Time**	6.979	**<0.001**
**Group*Time^2**	3.332	**0.010**
**Group*Sex**	1.395	0.235

Ingredient	Quantity
**Agar**	
**Water**	
Maize Flour	3600 g
Yeast Powder	732 g
Soya	432 g
**Molasses Mix**	
Malt	3600 g
Molasses	1050 g
Water	2 L
**Nipagin mix**	
Methyl 4-Hydroxybenzoate	148 g
Ethanol	1300 mL
Water	200 mL
**Acid Mix**	
Propionic acid	1 L
Orthophosphoric acid	64 mL

Standard fly food recipe –

Recipe for the standard fly food used in this study.

Supplementary References

1 Edward, D. A., Fricke, C., Gerrard, D. T. & Chapman, T. Quantifying the life-history response to increased male exposure in female *Drosophila melanogaster*. *Evolution* **65**, 564-573, doi:10.1111/j.1558-5646.2010.01151.x (2011).

2 R Development Core Team. R: A language and environment for statistical computing. *R. Foundation for Statistical Computing, Vienna, Austria.* http://www.R-project.org/ (2015).
